# Dynamic Region of Interest Selection in Remote Photoplethysmography: Proof-of-Concept Study

**DOI:** 10.2196/44575

**Published:** 2023-03-30

**Authors:** Adam Kiddle, Helen Barham, Simon Wegerif, Connie Petronzio

**Affiliations:** 1 Xim Ltd Southampton United Kingdom; 2 The Text Doctor Wantage United Kingdom

**Keywords:** vital sign, vital sign measurement, remote photoplethysmography, contactless vital sign measurement, region of interest (ROI), biomedical sensing, facial camera PPG, signal processing, machine learning, smart device, mobile app, algorithm, skin tone

## Abstract

**Background:**

Remote photoplethysmography (rPPG) can record vital signs (VSs) by detecting subtle changes in the light reflected from the skin. Lifelight (Xim Ltd) is a novel software being developed as a medical device for the contactless measurement of VSs using rPPG via integral cameras on smart devices. Research to date has focused on extracting the pulsatile VS from the raw signal, which can be influenced by factors such as ambient light, skin thickness, facial movements, and skin tone.

**Objective:**

This preliminary proof-of-concept study outlines a dynamic approach to rPPG signal processing wherein green channel signals from the most relevant areas of the face (the midface, comprising the cheeks, nose, and top of the lip) are optimized for each subject using tiling and aggregation (T&A) algorithms.

**Methods:**

High-resolution 60-second videos were recorded during the VISION-MD study. The midface was divided into 62 tiles of 20×20 pixels, and the signals from multiple tiles were evaluated using bespoke algorithms through weighting according to signal-to-noise ratio in the frequency domain (SNR-F) score or segmentation. Midface signals before and after T&A were categorized by a trained observer blinded to the data processing as 0 (high quality, suitable for algorithm training), 1 (suitable for algorithm testing), or 2 (inadequate quality). On secondary analysis, observer categories were compared for signals predicted to improve categories following T&A based on the SNR-F score. Observer ratings and SNR-F scores were also compared before and after T&A for Fitzpatrick skin tones 5 and 6, wherein rPPG is hampered by light absorption by melanin.

**Results:**

The analysis used 4310 videos recorded from 1315 participants. Category 2 and 1 signals had lower mean SNR-F scores than category 0 signals. T&A improved the mean SNR-F score using all algorithms. Depending on the algorithm, 18% (763/4212) to 31% (1306/4212) of signals improved by at least one category, with up to 10% (438/4212) improving into category 0, and 67% (2834/4212) to 79% (3337/4212) remaining in the same category. Importantly, 9% (396/4212) to 21% (875/4212) improved from category 2 (not usable) into category 1. All algorithms showed improvements. No more than 3% (137/4212) of signals were assigned to a lower-quality category following T&A. On secondary analysis, 62% of signals (32/52) were recategorized, as predicted from the SNR-F score. T&A improved SNR-F scores in darker skin tones; 41% of signals (151/369) improved from category 2 to 1 and 12% (44/369) from category 1 to 0.

**Conclusions:**

The T&A approach to dynamic region of interest selection improved signal quality, including in dark skin tones. The method was verified by comparison with a trained observer’s rating. T&A could overcome factors that compromise whole-face rPPG. This method’s performance in estimating VS is currently being assessed.

**Trial Registration:**

ClinicalTrials.gov NCT04763746; https://clinicaltrials.gov/ct2/show/NCT04763746

## Introduction

Measurement of vital signs (VSs) is an integral component of clinical monitoring and, importantly, enables early detection of clinical deterioration [1]. The COVID-19 pandemic has prompted interest in the use of remote technology to monitor patients with nonserious symptoms, reducing the burden on health care facilities and the risk of infection during visits to health care facilities [2]. Contactless technology is also potentially useful where current care cannot be readily used, such as in mental health settings [3].

**Figure 1:**
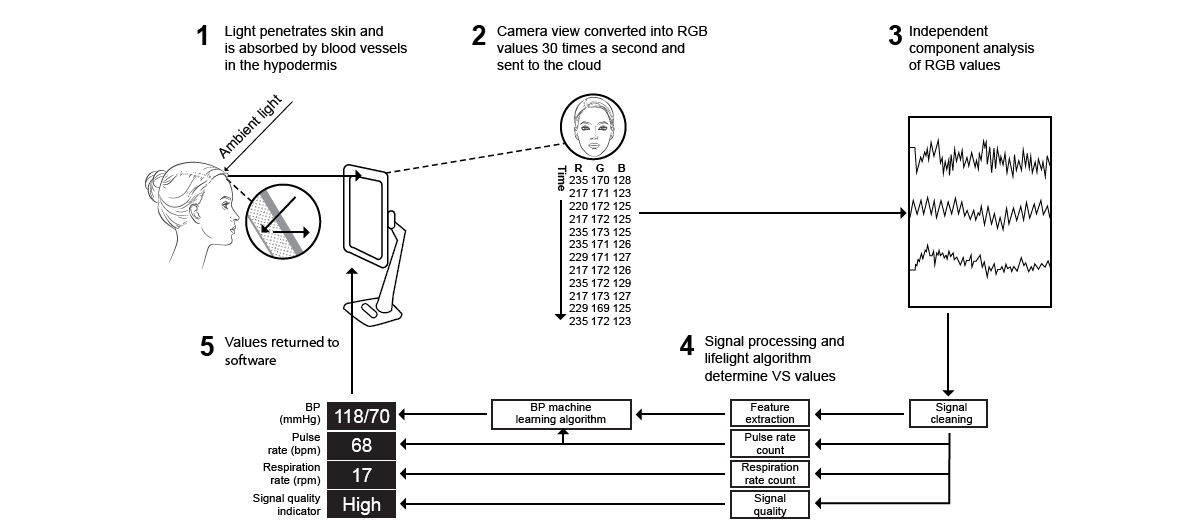
Use of remote photoplethysmography in the Lifelight software [11]. BP: blood pressure; RGB: red, green, blue; VS: vital sign. Figure adapted from the cited source which is published under Creative Commons Attribution 4.0 International License [12]).

Photoplethysmography has been used to measure pulse rate (PR) [4,5], arterial oxygen saturation [6], and respiratory rate (RR) [4,7], to estimate blood pressure (BP) [8,9], and to detect atrial fibrillation [10]. Remote photoplethysmography (rPPG) has the potential to measure VS by detecting changes in the light reflected from the skin surface (Figure 1, part 1).

Lifelight (Xim Ltd) is novel software being developed as a medical device for the contactless measurement of VS by rPPG based on live video capture of the face using the integral camera on smart devices (eg, laptops and smartphones; Figure 1, part 1). The software captures the average color of regions of interest (ROIs) 30 times every second for 60 seconds (Figure 1, part 2). The resultant pulse waveform is processed to determine VS (Figure 1, parts 3-5).

The accuracy of Lifelight in estimating PR, RR, and BP has been demonstrated in the VISION-D and VISION-V studies, the former based on 17,233 measurements from 8585 participants [13]. Data from these studies supported the certification of Lifelight as a Class 1 Conformité Européenne medical device [14].

To date, the development of rPPG has focused on signal extraction and computational methods. ROI is an important determinant of the rPPG signal [15], influencing signal quality and morphology and the computational load [16], but it is relatively unexplored in the literature. An evaluation of 7 rPPG methods found that 2 were based solely on face recognition, using as much of the face as possible, and 4 extracted only specific skin colors [15]. However, only a small proportion of the raw video signal generated by rPPG is the pulsatile signal required for the measurement of VS; the majority of the signal reports skin tone. Extensive signal extraction and analysis are therefore required to extract the relevant signal to derive VS, which can be computationally expensive.

Conventional methods based on the whole face assume that different parts contribute equally to the signal, whereas this is not the case because irrelevant information is included, such as the background and facial areas covered by hair or glasses, for example. Skin thickness is an important determinant of rPPG signal quality, as this influences the depth of the blood vessels and therefore the absorption and scattering of light by skin tissue (see Figure 1, part 1). A cadaver study reported that the average thickness of the epidermis across 38 facial regions ranged from about 30 to 60 µm and the dermis from 760 to 1970 µm [17]. Thus, diffuse reflection cannot be expected to be uniform across facial regions. The correlation between skin thickness and the number of pixels extracted is better where the skin is thinnest and in the regions with the largest area (the forehead and cheeks); smaller regions are also more susceptible to noise from light distortion and movement [15]. Variation in vasculature and perfusion across the face is particularly important in the measurement of VS by rPPG. The cheeks and forehead are computationally efficient for rPPG because of their large area and good-quality signal [16]. The infraorbital artery, which perfuses the cheek, is potentially a good candidate for rPPG because it has strong pulsatile blood flow [18] and is less sensitive than the forehead and mouth to acute physiological stimuli (eg, temperature, taste, and emotions). However, the infraorbital artery shows wide interindividual variation anatomically, with 5 distinct phenotypes based on the number of branches [19]. Blood flow in the lower forehead and bridge of the nose are supplied by the internal carotid artery, which is influenced by autoregulation of cerebral blood flow and thus may not accurately reflect systemic BP, whereas blood flow to the upper forehead, the tip of the nose, cheeks, lips, and chin originates from the external carotid artery, which is not influenced by cerebral autoregulation and is, therefore, more closely aligned with systemic blood flow [20]. However, signal quality from the nose, mouth, and chin shows large interperson variability [16]. Factors such as changes in facial expression [21] and movements such as blinking and talking also affect the rPPG signal [22]. Lighting is particularly important: light distribution and intensity vary with the position of the subject relative to the light source, resulting in different visible pulse waveforms in different facial regions [23]. Variations in ambient light from light sources can severely disturb the subtle color changes due to pulsatile flow [22].

**Figure 2:**
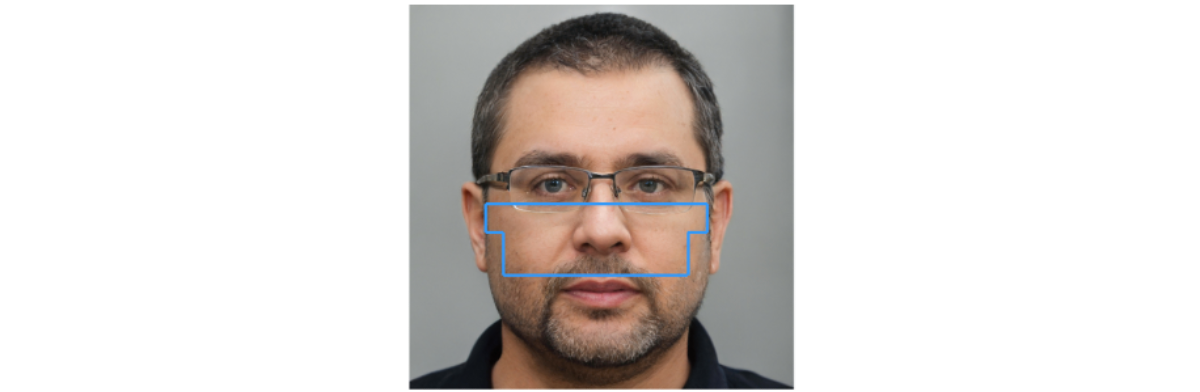
Computer-generated image showing the midface from which data were extracted (image generated using StyleGAN artificial intelligence [24]).

Thus, many factors influence signal quality in the measurement of VS using rPPG. In order to further develop Lifelight as a medical device suitable for routine clinical use, the software algorithms must be able to identify the optimal ROI in each subject: areas of the face where fluctuations in blood flow are most readily detected and measured, in order to provide a robust and reliable signal for the determination of BP. This is important for real-world applications where operational factors, such as the orientation of the face relative to the camera, are harder to control than in the laboratory. Following the VISION-V and VISION-D studies (which used the whole face), we refined the data processing to focus on the midface region, comprising the cheeks, nose, and top lip (Figure 2).

From early clinical studies, it became clear that signal quality was often compromised by movement and an inappropriate orientation of the face towards the camera. Here we describe a method for dynamic ROI selection in which the midface region is divided into small “tiles” and bespoke algorithms are used to identify and aggregate the optimal tiles in terms of signal quality for each subject. This tiling and aggregation (T&A) approach is verified by the evaluation of rPPG signal morphology by a trained observer. This is a widely accepted method for the validation of machine learning [25] and is analogous to the visual screening of other VS data, such as the assessment of electrocardiographs by trained electrophysiologists. As high-quality rPPG signals are more difficult to obtain from individuals with darker skin tones (eg, Fitzpatrick skin types 5 and 6) because of light absorption by melanin [26], we have also explored the performance of the T&A method across the Fitzpatrick skin tone range.

## Methods

### Video Recordings

Video recordings for this study were made during a cross-sectional observational study (VISION-MD; NCT04763746), which is evaluating Lifelight for the measurement of VS compared with current standard of care methods [27]. The study involves adults (aged ≥16 years) who are inpatients (including critically ill patients), outpatients, friends or relatives of patients, and members of hospital staff. There were no exclusion criteria in order to gather data from people with a wide range of health statuses.

VS (PR, RR, oxygen saturation, and BP) were measured by trained nursing staff and clinical trial assistants using standard of care equipment. A 60-second video of each participant’s face was recorded simultaneously using Lifelight data collection software (Data Collect, Xim Ltd) via the front camera on an Apple iPad (model generation 6, 7, or 8). Videos were recorded at 1280×720 pixels and 30 frames per second using an H-264 minimal compression algorithm. Trial staff were asked to position participants with even lighting on their faces, and participants were asked to stay as still as possible. Background luminosity was measured using a handheld lux meter.

Videos were saved to the internal storage of the iPad in encrypted form. Anonymized rPPG data (the average color of areas of the face) were saved immediately to a cloud server compliant with National Health Service Digital Technology Assessment Criteria [28] and Cyber Essentials certification [29]. Subsequent analysis was performed using the encrypted files, which were downloaded to a processing site, decrypted, and processed automatically (ie, without any person viewing the videos). This resulted in anonymized aggregated data sets.

### Ethics Approval

The study was conducted in accordance with Good Clinical Practice and was approved by the UK Health Research Authority (IRAS 289242). Participants either gave written informed consent or a consultee declaration form was completed for patients who lacked capacity. Data were handled and stored in accordance with current General Data Protection Regulations. As described above, all analyses were performed on anonymized data.

### Observer Assessment of Signal Quality

**Figure 3:**
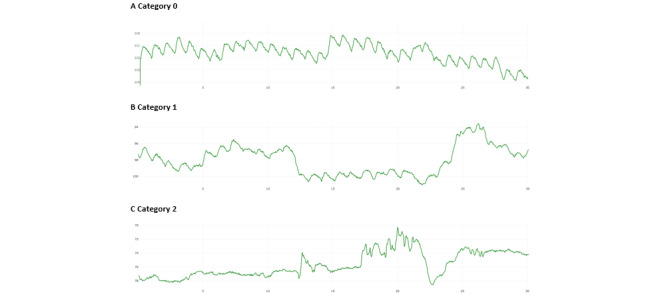
Examples of signals in each observer assessment category. All graphs are green intensity in analog to digital converter units versus time (s); descriptions are provided in Table 1.

To ensure that the improvement in SNR-F score represented a genuine improvement in signal quality, signals from the same videos were processed using the 2 different methods and assessed by an observer (CP) who was blinded to the prior signal processing. The observer method is more reliable than SNR-F for detecting signal features that are difficult to characterize using algorithms. The observer was trained to identify the key characteristics of the pulse waveform morphology that are important for the retrieval of physiological information. A custom signal visualizer tool developed by Xim Ltd was used, which runs in a web browser connected to a dedicated server over the internet. The observer analyzed the green light channel signal from the midface for the full 60-second recording and assigned each signal to one of 3 quality categories (0, 1, and 2, defined in Table 1). Example signals are shown in Figure 3. Raw signals, in which the troughs and crests of every pulse wave were evident with consistent amplitude and wavelength, were designated as “good” signals (category 0). These signals often had a high signal-to-noise ratio (SNR) and were used for model training. For signals that did not have a regular pulse wave formation, the observer determined whether the pulse decoding plot had picked up a constant frequency throughout; such signals were classified as category 1. Signals that did not have clear pulse waves were designated category 2. These videos were reviewed to determine the most likely reasons for poor quality, which were reported back to the clinical team to improve data collection (eg, to improve ambient lighting or minimize participant movement). The observer was blinded to the prior signal processing (ie, standard midface analysis or T&A).

**Table 1:** Categories used by the observer in the signal quality assessment (examples are shown in Figure 3).

Category	Quality	Description	Potential use
0	High	Clear and consistent waveform throughout	Suitable for algorithm training and development
1	Medium	Pulse visible for most of the signal but irregular	Suitable for algorithm testing
2	Poor	Irregular raw signal and no detectable pulse, or pulse obscured by nonphysiological noise, or baseline level shifts	Not suitable for development or testing without further processing

### T&A Approach

#### Overview

**Figure 4:**
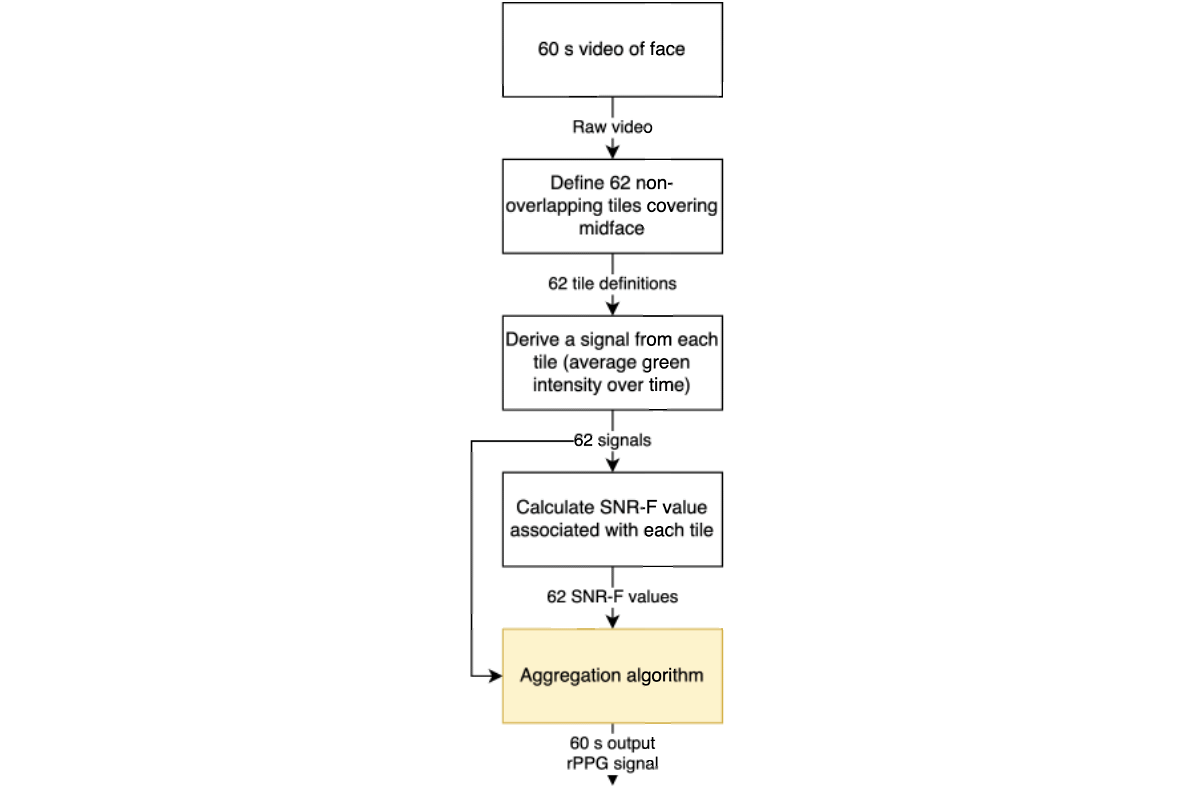
Video processing method. SNR-F: signal-to-noise ratio in the frequency domain; rPPG: remote photoplethysmography.

In each video frame, the midface area was divided into 62 tiles, each measuring 20×20 pixels, with no gaps or overlap. Average green light values were determined for all the tiles in each frame, and the signal quality was calculated for each tile (see section Determination of Signal Quality). The output signal was then derived based on the “best” tile signals (ie, the highest signal quality) using different aggregation algorithms. This method is illustrated in Figure 4.

#### Determination of Signal Quality

For tiling to be successful, all included tiles must be good candidates for the recovery of high-quality pulse data. The signal quality of each tile was determined from the SNR in the frequency domain (SNR-F) of the green channel, which gives the highest SNR due to absorbance from hemoglobin and oxyhemoglobin (compared with the blue and red channels) [30]. The signal power at the pulse frequency (PR in Hz ±0.15 Hz) was compared with the signal power in the remaining 0.5-5–Hz band.

#### Tile Aggregation

Several different methods for aggregating the signals from multiple tiles were tested, using bespoke algorithms based on weighting according to SNR-F score or segmentation (Table 2). Algorithms based on the “best 30 tiles” were used, as these incorporated about half of the midface area without being excessively computationally expensive.

**Table 2:** Methods used for tile aggregation.

Algorithm	Description
All tiles weighted	Takes all tile signals (weighted by each tile’s SNR-F score) and determines a weighted average
Best N tiles	Only the “best” N tiles (tiles with the highest SNR-F score) are averaged into 1 signal (eg, always taking the top 30 scoring tiles)
Best N tiles weighted	The best N tiles, weighted by the tile’s SNR-F score, are averaged into 1 signal
Best N tiles segmented^a^	Videos are cut into time-discrete segments; the tiles for each time segment are scored, and the best N tiles for that segment are combined; and multiple time segments may be combined or overlapped to smooth the signal and avoid sharp discontinuities at the boundaries of segments
Best N tiles segmented and weighted	A combination of the best N tiles weighted and best N tiles segmented algorithms

^a^In segmentation algorithms, a 60-second video is split into, for example, 6 segments of 10 seconds, and the “best N” tiles (in terms of SNR-F score) are identified and aggregated for each segment. Thus, different tiles may be selected for each 10-second segment. The aggregated signals retrieved for the segments are then recombined into a full 60-second output signal.

### Performance Assessment

To assess the performance of T&A, boundaries for the observer categories defined in Table 1 (0-2) were determined based on SNR-F scores. Thus, the SNR-F scores for all signals in 1 category were calculated, and the mean (*µ*) and standard deviation (*σ*) were determined. The boundaries between categories 2 and 1 (b_2,1_) and between categories 1 and 0 (b_1,0_) were estimated as follows:



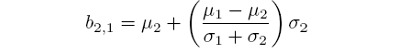





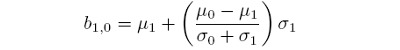



where *µ*_i_ and *σ*_I_ are the mean and standard deviation of the SNR-F scores for the signals in category i. A signal with an SNR-F score <b_2,1_ would be predicted as category 2; SNR-F≤b_2,1_ and <b_1,0_ would be predicted as category 1, and SNR-F≥b_1,0_ in category 0.

SNR-F scores for the midface analysis and following T&A were compared using quiver plots to determine whether the signal quality had improved sufficiently to justify promotion to a higher category (ie, from 1 to 0 or from 2 to 1 or 0). The quality categories assigned based on SNR-F scores were compared with the categories assigned by the observer, as described in the section Observer Assessment of Signal Quality.

In a secondary analysis, a set of signals that would be predicted to be promoted into a higher observer category following T&A based on SNR-F scores were identified and compared with the actual observer-assigned categories. Only signals with SNR-F scores that were at least 2 dB from the category boundaries (whether based on the midface or following T&A) were selected. This criterion minimized uncertainty about the estimated category, as 2 dB was approximately 20% of the distance between category boundaries.

### Effect of Skin Tone

Observer ratings and SNR-F scores were compared before and after T&A for Fitzpatrick skin types 5 and 6.

## Results

A total of 4310 high-resolution videos recorded from 1315 participants as part of the VISION-MD study were used in this analysis.

### Signal Quality

**Figure 5:**
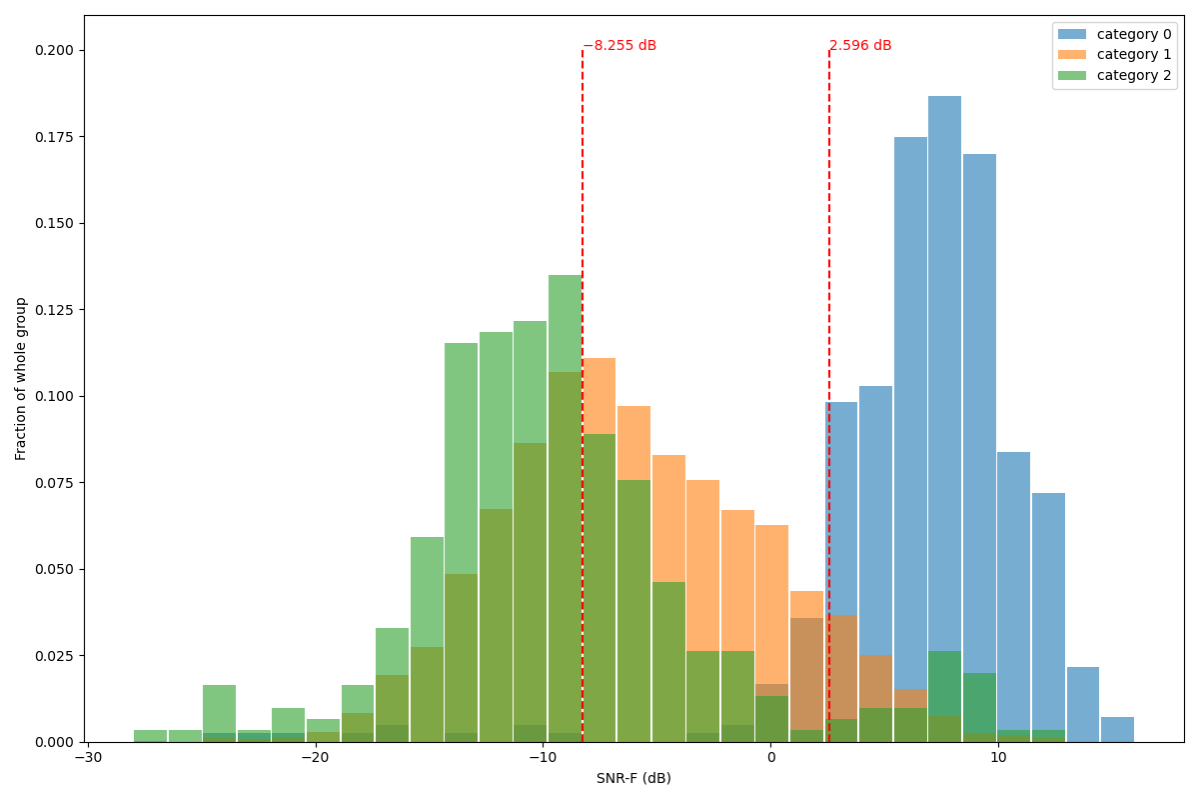
Distribution of observer ratings (4310 samples). The red dashed lines indicate observer category boundaries based on the signal-to-noise ratio in the frequency domain (SNR-F) scores.

Figure 5 shows the distribution of SNR-F scores for the 3 observer categories based on analysis of the midface region (ie, without T&A). Note that there is some overlap in SNR-F scores between categories because the assignments are based on the characteristics of the pulse, whereas SNR-F scores are a single value. Overall, signals with lower SNR-F scores fell largely within the poor-quality category (category 2), while those with higher SNR-F scores fell into category 0 (highest quality). Estimated boundaries between the categories based on SNR-F scores are also shown in the figure; it is evident that signals with SNR-F scores >2.596 dB were the most likely to fall into category 0.

### Effect of Tiling on Signal Quality

**Figure 6:**
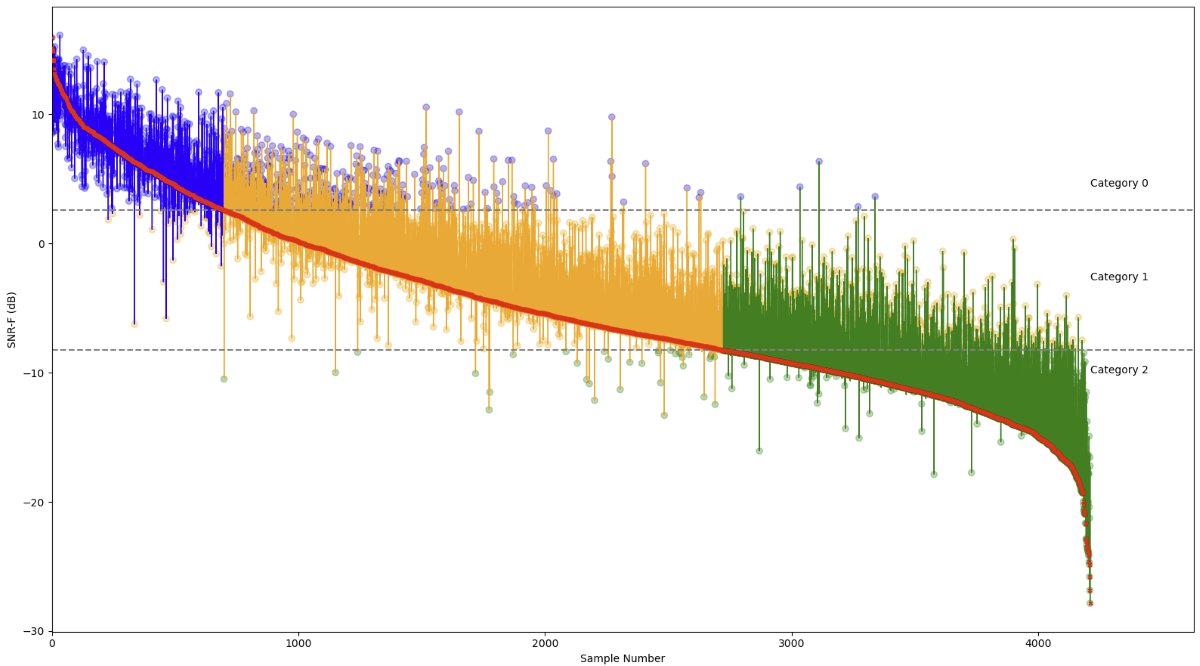
Quiver plot comparing signal-to-noise ratio in the frequency domain (SNR-F) signals before and after tiling and aggregation (T&A) using the “best 30 tiles segmented and weighted” algorithm. For each video, an arrow is plotted from the SNR-F score before T&A to the SNR-F score following T&A. The tests have been ranked so that increases and decreases in the score are seen as deviations from the central descending curve.

**Figure 7:**
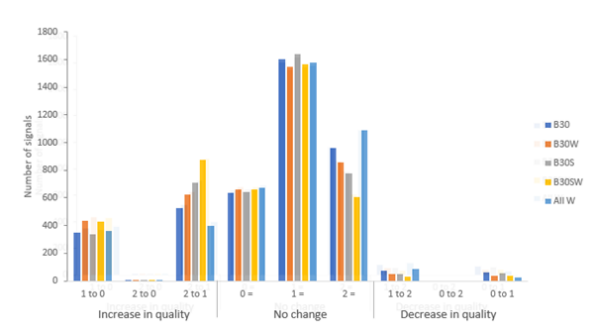
Changes in the signal quality category following tiling and aggregation. Values are the numbers of signals that changed category (0 [highest quality], 1, 2 [lowest quality]) following tiling and aggregation (= indicates that a signal remained in the same category). B: best; S: segmented; W: weighted.

The SNR-F scores from the midface analysis were compared with those following T&A for each video using quiver plots, as illustrated in Figure 6. T&A improved the mean SNR-F (Table 3). Depending on the algorithm, 18% (763/4212) to 31% (1306/4212) of signals improved by at least one category, with up to 10% (438/4212) improving into category 0, and 67% (2834/4212) to 79% (3337/4212) remaining in the same category. Importantly, 9% (396/4212) to 21% (875/4212) improved from category 2 (not usable) into category 1 (Figure 7). Improvements were seen with all the algorithms tested (Table 2). No more than 3% of signals (85-137/4212) were assigned to a lower-quality category following T&A.

In the secondary analysis, 52 signals were identified that were predicted to change observer category based on a comparison of the SNR-F scores before and after T&A. Most of these signals (32/52; 62%) showed the predicted recategorization following T&A (Table 4). The segmented weighted algorithm appeared least reliable in predicting observer recategorization (33%), whereas the other algorithms performed similarly (64%-100%).

**Table 3:** Performance of the tiling and aggregation algorithms compared with the standard no tiling analysis (n=4212 samples) based on the signal-to-noise ratio in the frequency domain score (dB).

Algorithm	Mean (SD)	Lowest	Highest
No tiling	−4.88 (6.99)	−27.85	15.93
All tiles weighted	−3.21 (7.44)	−27.11	16.28
Best 30 tiles	−2.99 (6.87)	−25.21	16.49
Best 30 tiles weighted	−2.37 (6.92)	−22.36	16.67
Best 30 tiles in segments	−2.60 (6.42)	−23.89	16.02
Best 30 tiles in segments weighted	−1.86 (6.28)	−21.25	16.12

**Table 4:** Category reassignment in the secondary analysis.

Algorithm	Number of tests^a^	Number recategorized as predicted, n (%)	Number remaining in same category, n (%)
All tiles weighted	3	3 (100)	0 (0)
Best 30 tiles	11	7 (64)	4 (36)
Best 30 tiles weighted	18	13 (72)	5 (28)
Best 30 tiles segmented	8	5 (63)	3 (37)
Best 30 tiles segmented and weighted	12	4 (33)	8 (67)
Total	52	32 (62)	20 (38)

^a^The number of tests differs between algorithms because some were less effective so fewer recordings met the 2 dB threshold rule (eg, all weighted).

### Effect of Skin Tone

SNR-F scores based on the midface region without tiling were lower for skin tones 5 and 6 than for paler tones (Table 5), approximately twice as many signals were in the observer category 2 (not suitable for analysis) and none were in category 0. The mean SNR-F scores improved with T&A. For skin tone 5, scores varied from −4.10 to −5.59 following T&A, depending on the algorithm used, compared with −7.03 with no tiling. For skin tone 6, values following T&A varied between −5.44 and −6.68, compared with −8.24 with no tiling. In addition, using the “best 30 tiles weighted” algorithm (which had the highest SNR-F score combined with appropriate waveform morphology in the analyses described above), 41% of signals (151/369) for skin tones 5 and 6 improved from observer category 2 to 1, and 12% (44/369) from category 1 to 0 (compared with 42% (1525/3632) and 22%, (436/3632) respectively, for skin tones 1-4).

**Table 5:** Signal quality by Fitzpatrick skin tone. Values are mean (SD) signal to noise ratio in the frequency domain scores (dB).

Skin tone	Sample size, n	No tiling	B^a^	BW^b^	BS^c^	BSW^d^	AW^e^
1	972	−2.63 (7.79)	−0.69 (7.55)	−0.06 (7.55)	−0.34 (7.08)	0.35 (6.86)	−0.71 (8.19)
2	1671	−4.97 (6.69)	−3.17 (6.56)	−2.53 (6.67)	−2.74 (6.1)	−1.99 (6.03)	−3.48 (7.16)
3	523	−5.64 (6.51)	−3.4 (6.47)	−2.84 (6.53)	−3.11 (6.03)	−2.39 (5.87)	−3.89 (6.88)
4	466	−5.60 (7.05)	−3.96 (6.78)	−3.28 (6.77)	−3.39 (6.29)	−2.58 (6.2)	−3.78 (7.47)
5	243	−7.03 (5.67)	−5.25 (5.71)	−4.74 (5.64)	−4.90 (5.26)	−4.10 (5.09)	−5.59 (6.03)
6	126	−8.24 (4.75)	−6.45 (5.02)	−5.92 (4.91)	−6.25 (4.43)	−5.44 (4.27)	−6.68 (5.66)

^a^B: best 30 tiles.

^b^BW: best 30 tiles weighted.

^c^BS: best 30 tiles segmented.

^d^BSW: best 30 tiles segmented and weighted.

^e^AW: all tiles weighted.

## Discussion

### Principal Findings

Including the whole face in the ROI introduces many variables and inconsistencies in the rPPG signal —the face is not a flat surface, and light reflection will differ because of the slightly different angles of incidence. Signal processing methods have been used to correct for, for example, fluctuations in illumination [22,31,32] and facial variation [21]. Lam and Kuno [32] and Song and colleagues [22] reported methods for ameliorating the effects of uneven light distribution through the selection of several small ROIs; however, the ROI cannot be too small because of potential interference from quantization noise [22]. We have taken a different approach in which we optimized ROI selection in order to obtain smaller amounts of the most relevant, high-quality data from each individual to reduce interference from external factors.

We based our method on the midface (Figure 2), where blood flow is most easily measured and large areas of irrelevant data are disregarded. However, this area is still subject to the influence of uneven lighting, facial expression, and the position of the subject—no matter how well a subject is positioned, it is difficult to ensure that the same ROI is captured every time. Our method of dynamic ROI selection using T&A overcomes many of the issues of a “static” fixed ROI, by identifying the highest quality signals for each subject. The T&A approach ignores areas of the face that are obscured (such as by glasses or facial hair, as in Figure 2). The midface was divided into 62 tiles of 20×20 pixels. Initial exploratory studies indicated that the size of the tile was not critical, although the largest and smallest tiles produced poor-quality signals. We used algorithms based on the “best 30 tiles,” as this incorporated about half of the midface area without being excessively computationally expensive. This proof-of-concept study demonstrates that T&A improves the signal quality in most cases compared with the entire midface, as evidenced by the improvements in the observer-rated category and increased SNR-F scores. Importantly, this approach also improved the signal quality for individuals with darker skin tones. T&A improved signal quality in Fitzpatrick skin tones 5 and 6 in terms of SNR-F score and observer-defined quality categories. Although the Fitzpatrick Skin Type Scale is the accepted method for defining skin tone [33], its use has been criticized because of racial bias, a weak correlation with skin color, and broad within-group variations in skin tone [33]. Spectro-colorimetry, which uses multiple variables to categorize skin tone objectively, has been proposed as an alternative [33], which may be incorporated into future studies.

To ensure that the improvement in SNR-F score represented a genuine improvement in signal quality, signals from the same videos were processed using the 2 different methods and assessed by an observer blinded to the prior signal processing (midface analysis vs T&A). Human observation is an accepted method for the task-based evaluation of medical images [25] and has been used to validate the machine-based assessment of, for example, atrial fibrillation [10], prostate cancer histology [34], and breast cancer diagnosis [35]. Independent annotator assessment has also been used to validate the signal quality of photoplethysmography signals recorded by mobile phones [36]. In our study, there was a good match between the signal quality category determined by the observer and SNR-F scores. Importantly, as further verification, signals that we predicted would be improved by T&A based on the SNR-F score were indeed assigned to a higher quality category by the observer, although a few signals had lower quality. This may arise for various reasons; for example, some tiles may contain high-quality signals for only part of the recording period (which is compensated for by the segmented approach to aggregation), or external noise may be interpreted as a pulse waveform if it has the right frequency (~1 Hz).

In this study, SNR-F was used as the metric for signal quality and performed well when compared with the observer-determined quality category. The SNR-F was able to capture both a reduction in noise and a strengthening of the pulsatile signal content. It may be possible to develop bespoke signal quality metrics optimized for specific VS, allowing emphasis on the quality of the most relevant aspects of the pulsatile signal for a given VS.

We tested 5 different algorithms, all of which improved the signal quality compared with using the whole midface. The “all tiles aggregated” method performed worst, demonstrating the value of focusing on fewer tiles with the best signal. While the “segmented” approach appears to perform better in terms of SNR-F score, our secondary analysis found that signals were not promoted into the expected higher observer category when using this method. Some of these signals showed marked baseline drift and switching the tiles of interest with time appeared to act destructively on the signal. Thus, while using the SNR-F score as the quality metric suggests that segmenting is a useful approach, the accepted method of observer rating based on the signal morphology indicated otherwise, underlining the importance of observer rating in the assessment. The current study aimed to demonstrate the concept of T&A rather than specifically comparing different algorithms. Future studies will continue to explore the optimal aggregation approach for each VS.

This preliminary study was based on a large amount of data collected in a clinical setting rather than under tightly controlled laboratory conditions, thus allowing for issues typically encountered in the clinical measurement of VS using rPPG. The study has high external validity because it collected data in real-world settings in which the Lifelight software will be used. In addition, different ways to aggregate tile signals were explored systematically, and their effects were compared against the accepted method of human observation. We did not vary the size of the midface region, but this is unlikely to improve the method because the “best signal” tiles were rarely near the periphery of this area. Assessment by 1 observer ensures a consistent approach but introduces subjectivity and may risk distribution skewness, particularly in category 1. The observer was specifically trained to identify the most important features of the waveform, to maximize consistency. However, intraobserver reliability was not specifically measured in this proof-of-concept study. Future studies will involve independent assessment by more than one observer, followed by a discussion to reach a consensus and measurement of intraobserver variability. In addition, features or models that increase dimensionality (such as kernel methods–based machine learning or support vector machines) could be used to improve the separation between the categories.

Dynamic ROI detection represents a paradigm shift in rPPG by focusing on the collection of small amounts of high-quality data that most faithfully represent the pulse wave morphology in each individual rather than capturing a large amount of low-quality data. Rather than predefining a specific area of the face, our method ensures that the best signal is used for each subject. This approach also improves the quality of the rPPG signals from individuals with darker skin tones. It can also reasonably be expected to overcome issues such as uneven light distribution, given that the face is not a flat surface and that there is variation in the position and orientation of the face relative to the camera. Further studies will address these expectations. In addition, the T&A method may overcome issues caused by movement. Casalino and colleagues [37] have described a method for measurement of oxygen saturation using rPPG in which 3 ROIs are used for measurement and a fourth to track head movements. We are exploring the impact of movement on rPPG, whether this differs between healthy volunteers and sick patients, and how T&A may address issues caused by movement during the video recording.

### Conclusions

This proof-of-concept study demonstrates that dynamic ROI selection using T&A improves the quality of rPPG green channel signals, including in dark skin tones. The method was verified by comparison with a trained observer’s rating. T&A can reasonably be expected to overcome factors that compromise whole-face rPPG. Future studies will identify the optimal method for T&A for each VS.

## Abbreviations

BP: blood pressure
PR: pulse rate
ROI: region of interest
rPPG: remote photoplethysmography
RR: respiratory rate
SNR: signal-to-noise ratio
SNR-F: signal-to-noise ratio in the frequency domain
T&A: tiling and aggregation
VS: vital sign

## Data Availability

The data sets generated during and/or analyzed during the current study are not publicly available due to the commercial sensitivity of these data, which is mandated in Xim’s contractual obligations with the National Institute for Health Research and National Health Service user experience (NHSX), which funded this research. Moreover, the informed consent provided by study participants was made on the basis that only authorized individuals of the research team based at the study sites (Xim and Xim’s authorized partners) would have access to their data, even in anonymized form. Excluding other reasons for data access, the study data are available from the corresponding author upon reasonable request. A patent application for the methodology described in this paper was made to the United States Patent and Trademark Office on November 4, 2022 (Application 18/052, 728).
